# Ang II acutely stimulates Na,K‐pump in cells from proximal tubules by increasing its phosphorylation at S938 via a PI3K/AKT pathway

**DOI:** 10.14814/phy2.15508

**Published:** 2022-11-14

**Authors:** Fadia S. Hanna, Samaa Alkhouri, Carthic Rajagopalan, Kyungmin Ji, Raymond R. Mattingly, Douglas R. Yingst

**Affiliations:** ^1^ Department of Physiology Wayne State University, School of Medicine Detroit Michigan USA; ^2^ Department of Pharmacology Wayne State University, School of Medicine Detroit Michigan USA; ^3^ Present address: Department of Pharmacology & Toxicology Brody School of Medicine, East Carolina University Greenville North Carolina USA

**Keywords:** angiotensin II, Na,K‐pump, phosphorylation, proximal tubule, signaling

## Abstract

Angiotensin II (Ang II)‐dependent stimulation of the AT_1_ receptor in proximal tubules increases sodium reabsorption and blood pressure. Reabsorption is driven by the Na,K‐pump that is acutely stimulated by Ang II, which requires phosphorylation of serine‐938 (S938). This site is present in humans and only known to phosphorylated by PKA. Yet, activation of AT_1_ decreases cAMP required to activate PKA and inhibiting PKA does not block Ang II‐dependent phosphorylation of S938. We tested the hypothesis that Ang II‐dependent activation is mediated via increased phosphorylation at S938 through a PI3K/AKT‐dependent pathway. Experiments were conducted using opossum kidney cells, a proximal tubule cell line, stably co‐expressing the AT_1_ receptor and either the wild‐type (α‐1.wild‐type) or an alanine substituted (α‐1.S938A) form of rat kidney Na,K‐pump. A 5‐min exposure to 10 pM Ang II significantly activated Na,K‐pump activity (56%) measured as short‐circuit current across polarized α‐1.wild‐type cells. Wortmannin, at a concentration that selectively inhibits PI3K, blocked that Ang II‐dependent activation. Ang II did not stimulate Na,K‐pump activity in α‐1.S938A cells. Ang II at 10 and 100 pM increased phosphorylation at S938 in α‐1.wild‐type cells measured in whole cell lysates. The increase was inhibited by wortmannin plus H‐89, an inhibitor of PKA, not by either alone. Ang II activated AKT inhibited by wortmannin, not H‐89. These data support our hypothesis and show that Ang II‐dependent phosphorylation at S938 stimulates Na,K‐pump activity and transcellular sodium transport.

## Graphical abstract

Angiotensin II‐dependent stimulation of the rat kidney Na,K‐pump in proximal tubules is mediated at least in part via an increase in the phosphorylation of the Na,K‐pump at S938 through a PI3K/AKT(PKB)‐dependent pathway, not by PKA. S938 is a conserved site of phosphorylation on all four isoforms of the human Na,K‐pump and was thought to be exclusively phosphorylated by PKA. The Na,K‐pump is the active transport mechanism that drives sodium reabsorption in the proximal tubule, which increases blood pressure. Therefore, the identification of this signaling pathway to phosphorylate the Na,K‐pump at S938 in response to angiotensin II is potentially relevant to human physiology.

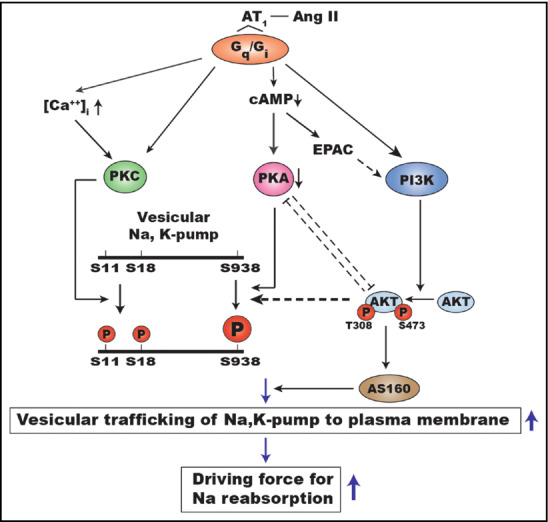

## 1 INTRODUCTION

Angiotensin II‐dependent stimulation of sodium reabsorption in the proximal tubule is energetically driven by the Na,K‐pump in the basolateral membrane, which increases blood pressure (Gurley et al., 2011; Li et al., 2011). It was initially thought that the acute stimulatory effect of Ang II on Na,K‐pump activity was only indirect (Brock et al., 1982): Ang II rapidly and directly stimulated NHE3 in the apical membrane (Geibel et al., 1990) to increase entry of sodium into the cell down its electrochemical gradient, elevating the concentration of intracellular sodium, which is the Na,K‐pump's rate‐limiting substrate. Later it was demonstrated in cultured opossum kidney (OK) cells, a proximal tubule cell line expressing the rat α‐1 subunit of the Na,K‐ATPase that Ang II acutely stimulates Na,K‐pump activity by rapidly increasing the amount of Na,K‐pump in the plasma membrane by a trafficking mechanism mediated by PKC‐dependent phosphorylation of the Na,K‐pump at S11 and S18 (Efendiev et al., 2003). In terms of relevance to human physiology, it is important to appreciate that S18 is unique to rodents and there are questions as to whether S11 is phosphorylated by PKC in humans (Poulsen et al., 2010). Also, these experiments were performed on cells grown on plastic, on which the cells do not develop distinct apical and basolateral polarity. Thus, it was not shown that Ang II‐dependent trafficking of the Na,K‐pump to the plasma membrane stimulated the net transport of sodium from the apical to basolateral side of the cell that occurs during sodium reabsorption.

We later demonstrated in OK cells stably co‐expressing the rat AT_1_ receptor and the rat α‐1 subunit of the Na,K‐pump, and also grown on plastic, that phosphorylation of the Na‐K pump at S938^1^ is also required for acute Ang II‐dependent stimulation of Na,K‐pump activity and increased trafficking of the Na,K‐pump to the plasma membrane (Massey et al., 2016). S938 is a site of PKA phosphorylation on all human forms of the Na,K‐pump (Poulsen et al., 2010), which suggests that Ang II‐dependent phosphorylation of S938 could be significant for human physiology. On the other hand, the idea that PKA‐dependent phosphorylation of S938 was required was a surprise, because the binding of Ang II to OK cells stably expressing the AT_1_ receptor markedly decreases intracellular concentrations of cAMP (Thekkumkara et al., 1998), which is the intracellular second messenger that directly activates PKA. Also, in our earlier studies we found that Ang II dependent phosphorylation of the Na,K‐pump at S938 is not blocked by H‐89 (Massey et al., 2012), which selectively inhibits PKA at the concentration used in our experiments. Thus, we investigated whether S938 could be phosphorylated by another kinase.

In considering how S938 could be phosphorylated in response to Ang II, we knew that PI3K was part of the mechanism by which Ang II stimulated the Na,K‐pump in smooth muscle (Isenovic et al., 2004). We also were aware that wortmannin, a selective inhibitor of PI3K at concentrations that do not appreciably affect PKA (Bain et al., 2007), regulates the trafficking of the Na,K‐ATPase (Yudowski et al., 2000) and that PI3K is known to activate AKT. The residues surrounding S938 are consistent with a consensus substrate motif for phosphorylation by AKT (Obata et al., 2000). Therefore, in this study we tested the hypothesis that acute Ang II‐dependent activation of the rat kidney‐pump in the proximal tubule is mediated via increased phosphorylation of the Na,K‐pump at S938 via a PI3K/AKT(PKB)‐dependent pathway.

## 2 MATERIALS AND METHODS

### 2.1 Cell lines

Two OK cell lines that stably co‐express the rat AT_1_ receptor and either the wild‐type (α‐1.wild‐type, clone 10C1.5) or S938A mutant (α‐1.S938A cells, clone C1.2) form of the rat kidney Na,K‐pump were developed and characterized by us over 10 years ago (Massey et al., 2012, 2016). For those two new cell lines we used an OK cell line stably expressing the rat AT_1_ receptor from the University of Colorado Health Sciences Center that had been previously characterized, a kind gift from Dr. Thekkumkara (Thekkumkara et al., 1998). As our study only involved cultured cells, it was exempt from institutional approval at Wayne State University. Cell cultures were maintained in DMEM‐F12 (HyClone) supplemented with 10% fetal bovine serum (Corning), 1% penicillin–streptomycin (Gibco) and 1 μM ouabain (Sigma) (Massey et al., 2012, 2016), which was included to kill any cells that were not primarily expressing the ouabain‐resistant rat α‐1 subunit of the Na,K‐pump. For immunoblotting experiments, cultures were switched to DMEM‐F12 without serum or ouabain supplementation on the day prior to stimulation and preparation of cell lysates (Massey et al., 2016; Yingst et al., 2008). For growth of cells on permeable supports, 100,000 cells were plated in medium without ouabain supplementation per filter [Costar Transwell #3470: 6.55‐mm inserts with polystyrene membrane and 0.4‐μm pore size (Corning)]. Cultures were used when the trans‐epithelial resistance was 300 ohms.

### 2.2 Measurement of Na,K‐pump activity

Na,K‐pump activity was measured as ouabain‐sensitive short‐circuit current (Isc) on Transwell plates as described in the results section. The approach used was similar to that previously developed (Gomes & Soares‐da‐Silva, 2002) except that we used nystatin (Cass et al., 1970) rather than amphotericin b to increase the apical permeability to sodium. The experiments were performed using a Voltage/Current Clamp (model VCC MC8), diffusion chambers (Diffusion Chamber P2300), and Easy Mount Diffusion Chamber Holders from Physiological Instruments. The chambers were kept at 37° C by means of a water bath from Thermo Scientific.

**FIGURE 1:**
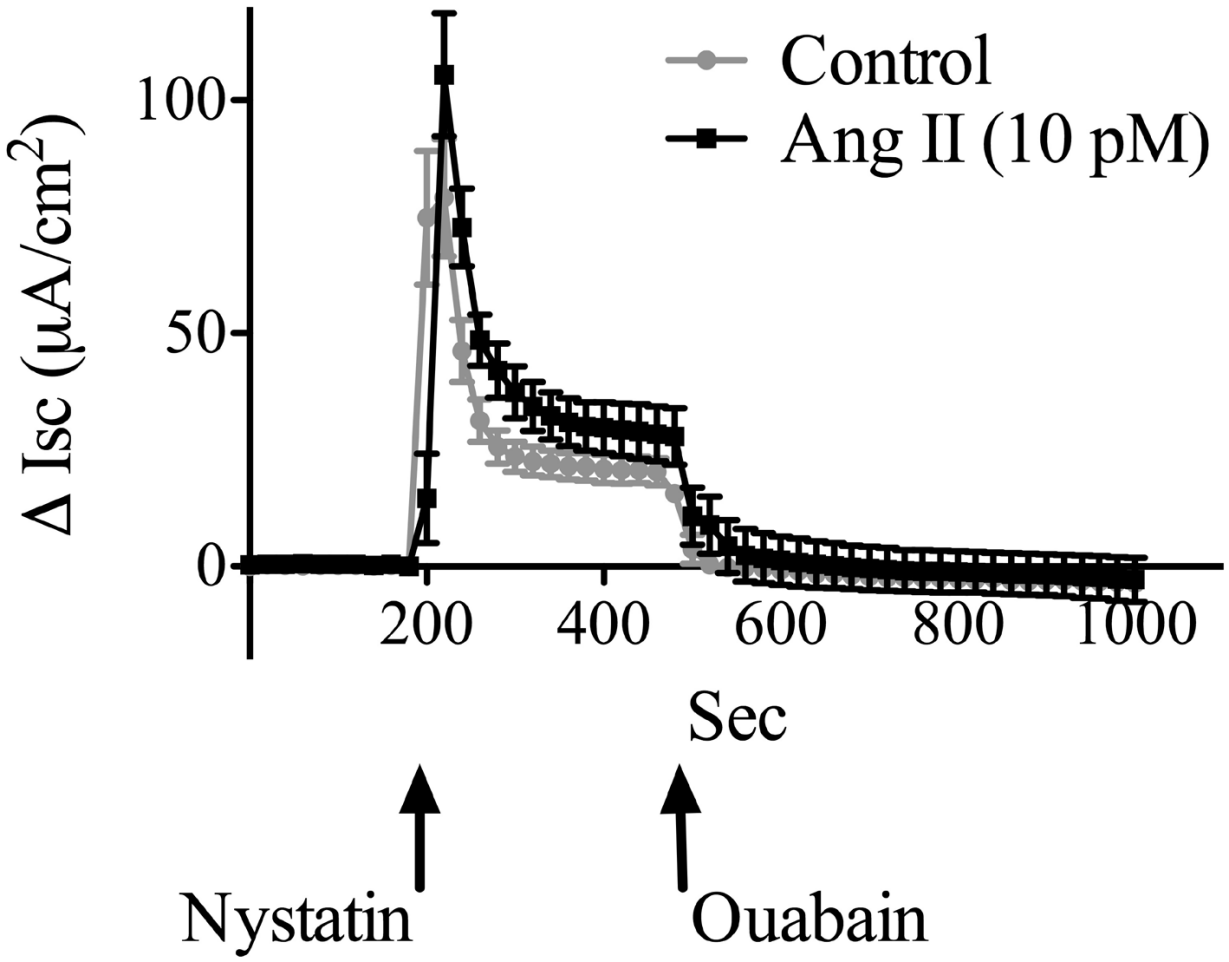
The effect of nystatin and ouabain on the change in short‐circuit current across confluent and polarized α‐1.wild‐type cells in the presence and absence of 10 pM ang II. The sodium ionophore nystatin was added to the apical membrane and ouabain, a specific inhibitor of the Na,K‐pump, was added to the basolateral membrane when shown. Ang II or vehicle (control) was added to the basolateral membrane 5 min before the nystatin (data shown are mean ± SEM; *n* = 6).

In each experiment, ~4 ml of our standard solution was placed on each site of the diffusion chambers. The liquid resistance and electrode offset potential between the two sides of each of the chambers was then adjusted to zero. Then Transwell plates containing either α‐1.wild‐type or α‐1.S938A cells were mounted in the chambers with ~4 ml of standard solution on each side of the cells. The standard solution contained 130 mM NaCl, 6 mM KCl, 0.7 mM MgCl_2_, 4.6 mM NaHCO_3_, 15 mM HEPES, 0.29 mM NaH_2_PO_4_, 1.3 mM Na_2_HPO_4_, 3.0 mM CaCl_2_, and 11 mM glucose at pH 7.4. Both sides of the cells were bubbled with 95% oxygen and 5% carbon dioxide and allowed to stabilize for 20 min. Immediately thereafter either buffer alone or buffer containing Ang II (Sigma Chemical Co.) to give a final concentration of 1, 10, or 100 pM was added to the basolateral side of the cells. An equal volume of buffer alone was added to the apical side of the cells. Five minutes later nystatin at a final concentration of ~595 USP Units/ml (Halevy et al., 1987) was added to the apical side to increase its permeability to sodium (Cass et al., 1970). An equal volume (20 μl) of DMSO was added to the basolateral side. Five min later 200 μl of 12 mM ouabain was added to the basolateral side of the cells (final concentration of 0.48 mM) and an equal volume of buffer was added to the apical side and the Isc was measured for an additional 15 min. The experiments to test the effects of wortmannin were carried out in a similar manner except that the initial equilibration period in buffer alone was 10 min, instead of 20 min. Then either 4 μl of DMSO or 4 μl of wortmannin (Calbiochem, San Diego, CA) in DMSO to give a final concentration of 0.2 μM was added to both sides. The cells were then incubated for an additional 10 min. Then either Ang II in buffer or buffer alone was added to the basolateral side of the cell and an equal volume of buffer alone was added to the apical side to give the indicated final concentrations. If the basolateral side of the cells were preincubated for 15 min in the standard solution containing 0.48 mM ouabain, the addition of nystatin increased the short‐circuit current <5% of that shown in the control in Figure 1.

### 2.3 Measurement of intracellular sodium concentration

The concentration of intracellular sodium in α‐1.wild‐type cells after the addition of nystatin was estimated from the change in the fluorescence of incorporated Asante NaTRIUM Green (TEF Labs, Inc.) (Roder & Hille, 2014). Measurements were made using a LEICA SP5 confocal microscope with an Argon laser set up to record images from live cells. Cells were maintained at 37° C in an atmosphere containing 5% CO_2_. The objective was a HCX PL APO CS 40.0x1.25 OIL UV, Refraction index 1.52, emission bandwidth PMT 2. The excitation wavelength was 514 nm. Emission was collected at 530 to 590 nm. During the measurements the power and gain were held constant.

A new stock concentration of Asante NaTRIUM Green was prepared for each experiment. A volume of 100 μl DMSO was added to 50 μg of Asante NaTRIUM Green and vortexed. This volume was then added to 10 ml of our standard solution to give a final concentration of 4.6 μM Asante NaTRIUM Green. After preparing the dye α‐1.wild‐type cells grown on Transwell plates were washed three times with the standard solution. Then the standard solution containing 4.6 μM Asante NaTRIUM Green was added to both sides of the cells and incubated for 45 min at 37°C. The dye was removed by aspiration and the cells washed three times. They were then incubated a second time for 20 min in our standard solution without dye. The cells were then washed one more time and immediately placed on the microscope with 150 μl standard solution on both the apical and basolateral sides of the cells. The fluorescence was then measured for 100 s. The plate was removed from the microscope and nystatin (Sigma Chemical Co.) was added to the apical side of the cells. The plate was put back in the microscope and the fluorescence was measured for 5 min. The time between the addition of the nystatin solution and the beginning of the live confocal recording was approximately 1 min.

At the end of the 5‐min period with nystatin the response of the dye as a function of intracellular sodium concentration was calibrated. First, the standard solution on the apical and basolateral side of the cell were removed by aspiration. Then both the apical and basolateral sides of the cells were washed three times with a solution containing 71 mM NaCl, 71 mM KCl, 0.7 mM MgCl_2_, 15 mM Hepes free acid, 3.0 mM CaCl_2_ and 5 μM gramicidin (Sigma Chemical Co.), pH 7.4. The gramicidin forms channels in the plasma membrane equally permeable to sodium and potassium (Busath, 1993). The plate was then put back on the microscope and the fluorescence measured. This solution was then removed by aspiration and same procedure, including washing three times with the next solution, was repeated for each of the five calibration solutions each containing 5 μM gramicidin. The next solution was made to contain 40 mM NaCl, 71 mM KCl, 0.7 mM MgCl_2_, 15.0 mM Hepes free acid, 3.0 mM CaCl_2_, and 31 mM choline Cl. The third was made to contain 10 mM NaCl, 71 mM KCl, 0.7 mM MgCl_2_, 15 mM Hepes free acid, 3.0 mM CaCl_2_, and 61 mM choline Cl, and the fourth to contain 0 mM NaCl, 71 mM KCl, 0.7 mM MgCl_2_, 15 mM Hepes free acid, 3.0 mM CaCl_2_, and 71 mM choline Cl, all at pH 7.4. The sodium concentrations in these solutions were measured by flame photometry and found not to be significantly different than the theoretical values, except that that the solution that ideally contained no sodium was measured to contain an average of 0.5 mM sodium.

In the calibration solutions itemized above the extracellular potassium concentration was increased compared to normal Ringer to minimize the loss of intracellular potassium in the presence of gramicidin. It was expected that the intracellular potassium would decrease somewhat but stabilize close to the concentration of 71 mM in the calibration solutions. In the presence of gramicidin, the plasma membrane of the OK cells should be about equally permeable to sodium and potassium. Therefore, the membrane potential of the cells should be much less negative than normal. Thus, the concentration of intracellular sodium should be only slightly greater than the concentration of extracellular sodium in our calibration solutions.

### 2.4 Phosphorylation experiments

Experiments were carried out on whole‐cell lysates as previously described (Massey et al., 2012, 2016). Relative phosphorylation is defined as the ratio of the amount of phosphorylation in the experimental sample compared to the control in that experiment, with each first corrected to the total amount of the protein of interest in that sample.

### 2.5 Materials

Anti‐P‐Ser‐473 Akt (#9271) and anti‐AKT antibodies (#9272) were from Cell Signaling Technology. The phosphospecific antibody to the α_1_‐isoform of the Na‐K pump that is phosphorylated at Ser^938^ (anti‐P‐Ser^938^) of the rat Na‐K‐ATPase α_1_‐isoform was developed by Cell Signaling Technology and previously characterized (Massey et al., 2016). The antibody against the *α*‐subunit of sheep kidney Na^+/^K^+^ ‐ATPase (M8‐P1‐A3) was purchased from Sigma–Aldrich. Secondary antibodies were from Jackson ImmunoResearch: donkey anti‐mouse (# 715‐035‐150) and anti‐rabbit (# 711‐035‐152) both coupled to horseradish peroxidase (HRP). H‐89 was from LC Laboratories. SDS‐PAGE reagents were from Fisher Scientific (Hanover Park); acrylamide was from Bio‐Rad Laboratories; polyvinylidene difluoride was from Millipore; KPL chemiluminescence reagents were from Insight Biotechnology; phosphatase inhibitors (microcystin and okadaic acid) were from Axxora; the molecular weight marker MagicMark was from Invitrogen. CPT‐2Me‐cAMP (CPT) was from Tocris. All other reagents were from Sigma‐Aldrich.

### 2.6 Statistical analysis

The results were analyzed by a one‐way ANOVA followed by Bonferroni's post‐hoc multiple comparison test using GraphPad Prism 5 software. Results were considered significant if *p* ≤ 0.05. In each experiment the value of *n* is equal to the number of independent experiments.

## 3 RESULTS

The direct effect of Ang II on the Na,K‐pump in the basolateral membrane of polarized proximal tubule cells was measured with the apical membrane made highly permeable to sodium to avoid confusing a direct effect of Ang II on Na,K‐pump activity with secondary activation. The latter is caused by Ang II first stimulating NHE3 in the apical membrane, increasing the concentration of intracellular sodium, which is the rate‐limiting substrate for the Na,K‐pump. The experimental basis for our approach to measure Na,K‐pump activity is introduced in Figure 1, which shows that the addition of nystatin, a sodium ionophore, to the apical membrane of polarized α‐1.wild‐type cells rapidly increased the short‐circuit current (Δ Isc) under control conditions. The Δ Isc reached a peak within a minute and then subsided to a sustained plateau value above the initial level (Figure 1, control). The subsequent addition of ouabain, a specific inhibitor of the Na,K‐pump, returned Isc to the level before nystatin (Figure 1). If 10 pM Ang II was added to the basolateral side of the cells 5 min before the addition of nystatin, the ouabain‐sensitive change in current was greater than under control conditions (Figure 1).

**FIGURE 2:**
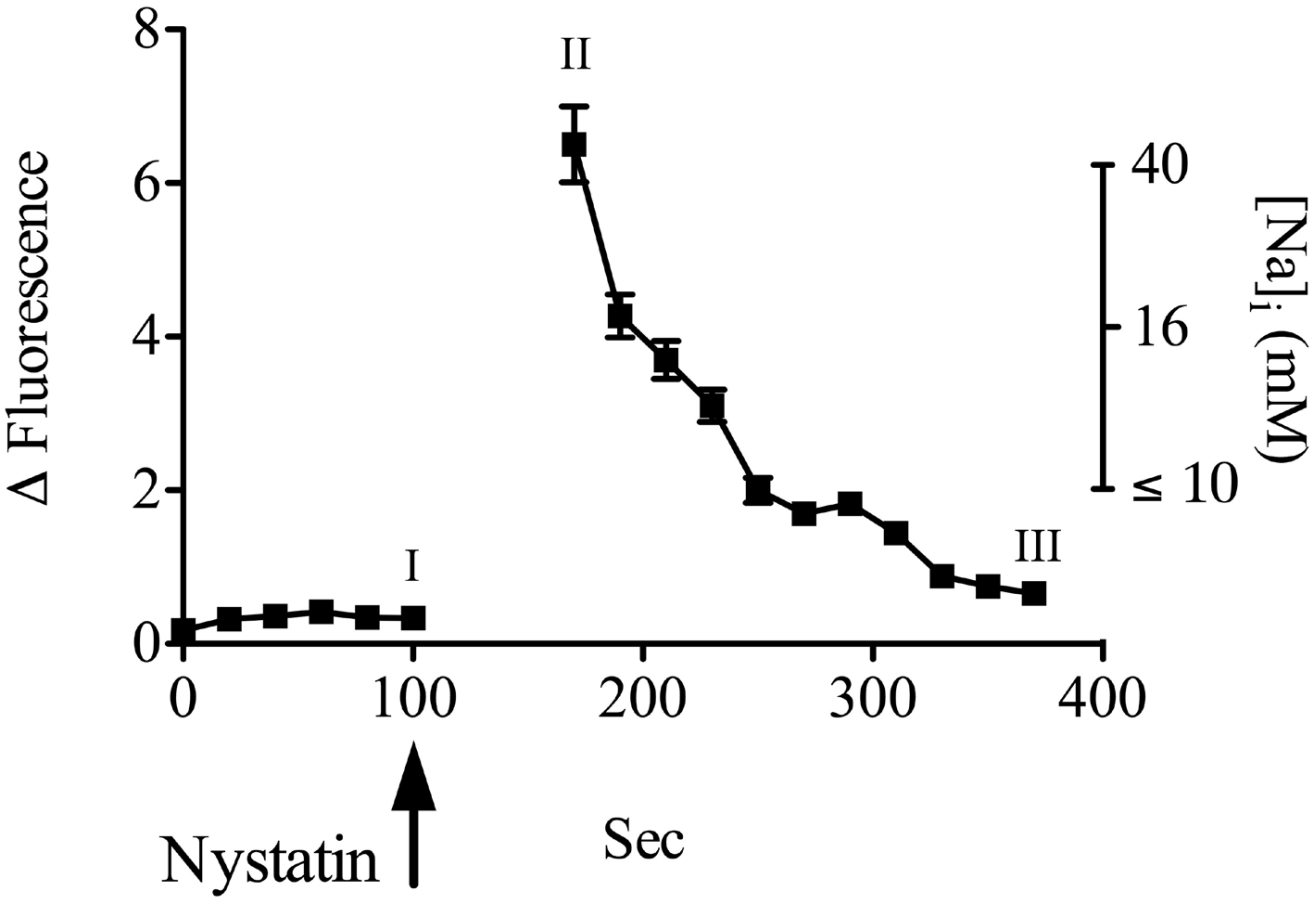
How the fluorescence of the sodium‐sensitive dye Asante NaTrium green inside α‐1.wild‐type cells changes in response to nystatin added to the apical membrane and the corresponding estimated intracellular sodium concentration. The fluorescence of the cells under control conditions is labeled “I.” The peak change in fluorescence after the addition of nystatin is labeled “II;” the minimum change above baseline after nystatin is labeled “III.” The corresponding intracellular sodium concentration on the right ordinate was calculated from changes in the fluorescence of Asante NaTrium green on a parallel Transwell plate using non‐linear least squares fit to a rectangular hyperbola, *B*_max_ = 9.1 mM, *K*_*d*_ = 21 mM; *y* = *B*_max_*X/[*K*_*d*_ + *X*]; *n* = 3.

We measured the concentration of intracellular sodium during the protocol used for the experiments shown in Figure 1. These measurements (Figure 2) confirmed that: nystatin increased the concentration of intracellular sodium as expected; and the initial peak in Isc that follows the addition of nystatin (see Figure 1) was correlated with elevated intracellular sodium. These results also inform why the Isc rapidly decreased after the initial peak following the addition of nystatin (see Figure 1). The addition of nystatin to the apical membrane in the absence of Ang II rapidly increased the concentration of intracellular sodium from resting levels (<10 mM) to over 40 mM (Figure 2). Over the next 5 min, the intracellular sodium concentration decreased to between 5 and 10 mM (Figure 2), which is close to the concentration that half‐maximally stimulates the Na,K‐pump under physiological conditions. Thus, the initial rise in Isc following the addition of nystatin is due to an increase in the concentration of intracellular sodium and the subsequent decline in Isc after nystatin is due to a subsequent decline in the concentration of intracellular sodium consistent with the cells containing active Na,K‐pumps.

Thus, the results in Figures 1 and 2 show that differentiated and confluent OK cells stably expressing the rat α‐1 subunit of the Na,K‐ATPase produce an ouabain‐sensitive net charge movement from the apical to the basolateral side of the cells when the apical membrane has been permeabilized to sodium using an ionophore for sodium. Accordingly, we have used the ouabain‐sensitive change in Isc that occurs upon the addition of nystatin and during the next 5 min, which is when ouabain is added, as a measure of Na,K‐pump activity.

**FIGURE 3:**
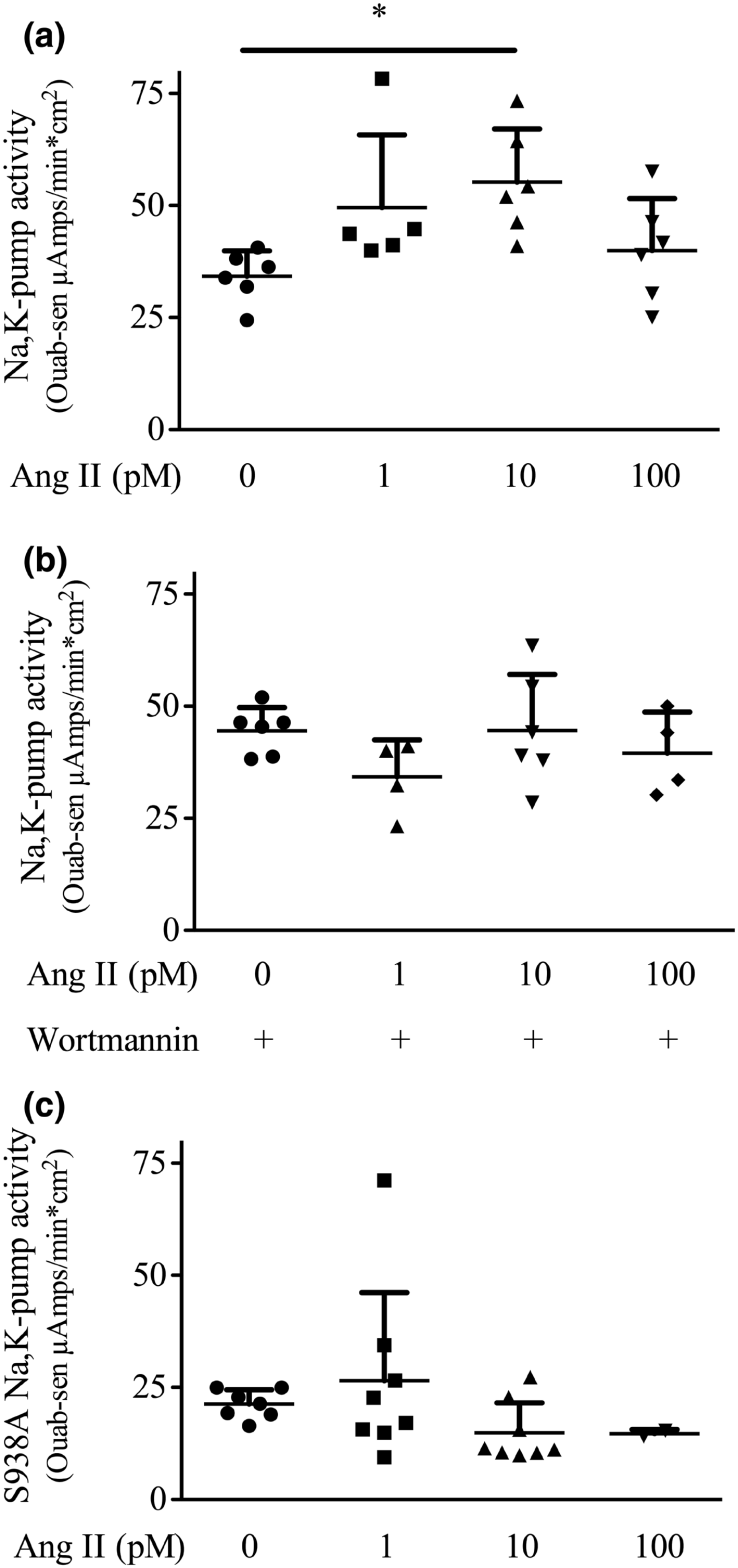
The effect of Ang II on Na,K‐pump activity. (a): α‐1.wild‐type cells; *n* = 6; (b): α‐1.wild‐type cells in the presence of 0.2 μM wortmannin; *n* = 6; (c): α‐1.S938A cells; *n* = 8, except for 100 pM Ang II, which is the mean of two experiments and was not included in the statistical analysis for the data in (c). All the data in (a) and (b) were included in the analysis. The horizontal lines are the median and interquartile range. The line labeled with “*” shows which samples are different from each other as analyzed by a one‐way ANOVA followed by Bonferroni's post‐hoc multiple comparison.

Adding Ang II 5 min before the addition of nystatin significantly stimulated Na,K‐pump activity in α‐1.wild‐type cells (Figure 3a). The post‐hoc analysis showed that the activity of the Na,K‐pump after stimulation with 10 pM Ang II was significantly different than the control (Figure 3a). In α‐1.wild‐type cells preincubated with 200 nM wortmannin, which is a selective inhibitor of PI3K that does not block PKA at 1 μM (Bain et al., 2007), Ang II had no effect on Na,K‐pump activity (Figure 3b). Ang II did not stimulate Na,K‐pump activity in α‐1.S938A cells (Figure 3c).

**FIGURE 4:**
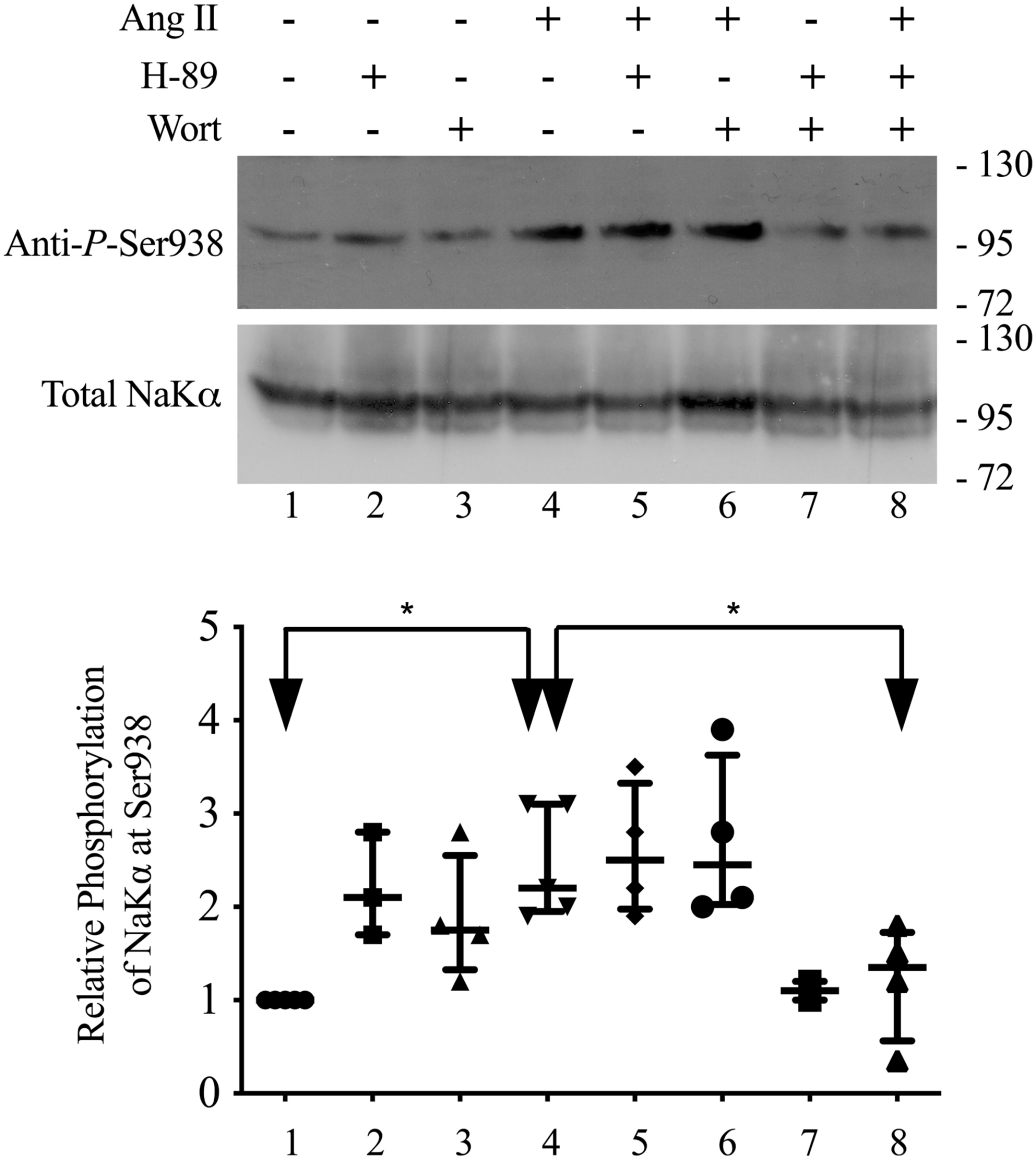
The effect of 10 μM H‐89 and 0.2 μM wortmannin on the ability of 100 pM Ang II to increase the phosphorylation of the Na,K‐pump at S938 in α‐1.wild‐type cells as measured in cell lysates; *n* = 4. The horizontal lines are the median and interquartile range. The top panel of the immunoblot shows the relative amount of phosphorylation of the Na,K‐pump at S938. The lower panel shows the relative amount of Na,K‐pump in each of the lines after the blot was stripped and reprobed with anti‐α. the cells were pre‐incubated with wortmannin and H‐89 for 20 min and then treated with Ang II for 2 min prior to sample preparation. The arrows labeled with “*” show which samples are different from each other as analyzed by a one‐way ANOVA followed by Bonferroni's post‐hoc multiple comparison.

**FIGURE 5:**
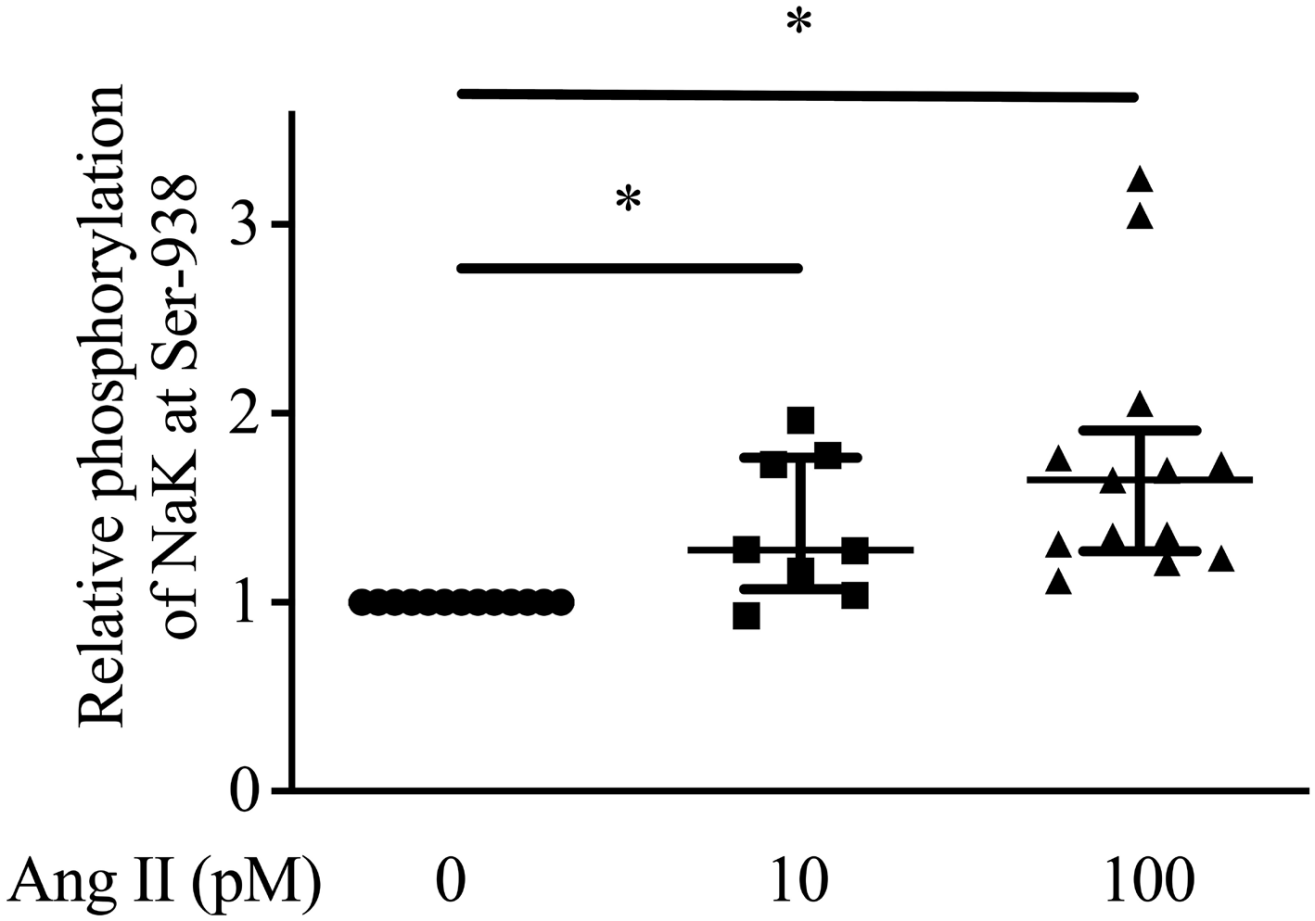
The relative effect of different concentrations of Ang II on the phosphorylation of the Na,K‐pump at S938 in α‐1.wild‐type cells as measured in cell lysates; *n* = 8. The horizontal lines are the median and interquartile range. The lines labeled with “*” show which samples are different from each other as analyzed by a one‐way ANOVA followed by Bonferroni's post‐hoc multiple comparison.

Incubating α‐1.wild‐type cells with Ang II significantly increased the phosphorylation of the‐pump at S938 as measured in whole cell lysates compared to control levels (Figure 4, lane 1 vs. lane 4, as shown by the double headed arrow with “*”). Both 10 and 100 pM Ang II significantly increased phosphorylation at S938 (Figure 5). Ang II‐dependent increases in the phosphorylation of the Na,K‐pump at S938 in α‐1.wild‐type cells (Figure 4, lane 1 vs. lane 4), were significantly reduced by the combination of H‐89 and wortmannin (Figure 4, lane 4 vs. lane 8, as shown by the double headed arrow with the “*”), not by either H‐89 (Figure 4, lane 4 vs. 5) or wortmannin alone (Figure 4, lane 4 vs. 6) as analyzed by a one‐way ANOVA followed by Bonferroni's post‐hoc multiple comparison. In addition, H‐89 increased the phosphorylation of S938 compared to control (Figure 4, lane 2 vs. lane 1; *p* = 0.03) and wortmannin increased the phosphorylation of S938 compared to control (Figure 4, lane 3 vs. lane 1; *p* = 0.035) as analyzed by a paired t‐test. Likewise, Ang II increased the phosphorylation at S938 in the presence of H‐89 compared to control (Figure 4, lane 5 vs. lane 1; *p* = 0.01) and increased the phosphorylation at S938 in the presence of wortmannin compared to control (Figure 4, lane 6 vs. lane 1; *p* = 0.015), and the combination of H‐89 and wortmannin inhibited the phosphorylation of S938 observed in the presence of Ang II and H‐89 (Figure 4, lane 8 vs. lane 5; *p* = 0.029). A negative control for anti‐P‐Ser938 was presented previously (Massey et al., 2012).

**FIGURE 6:**
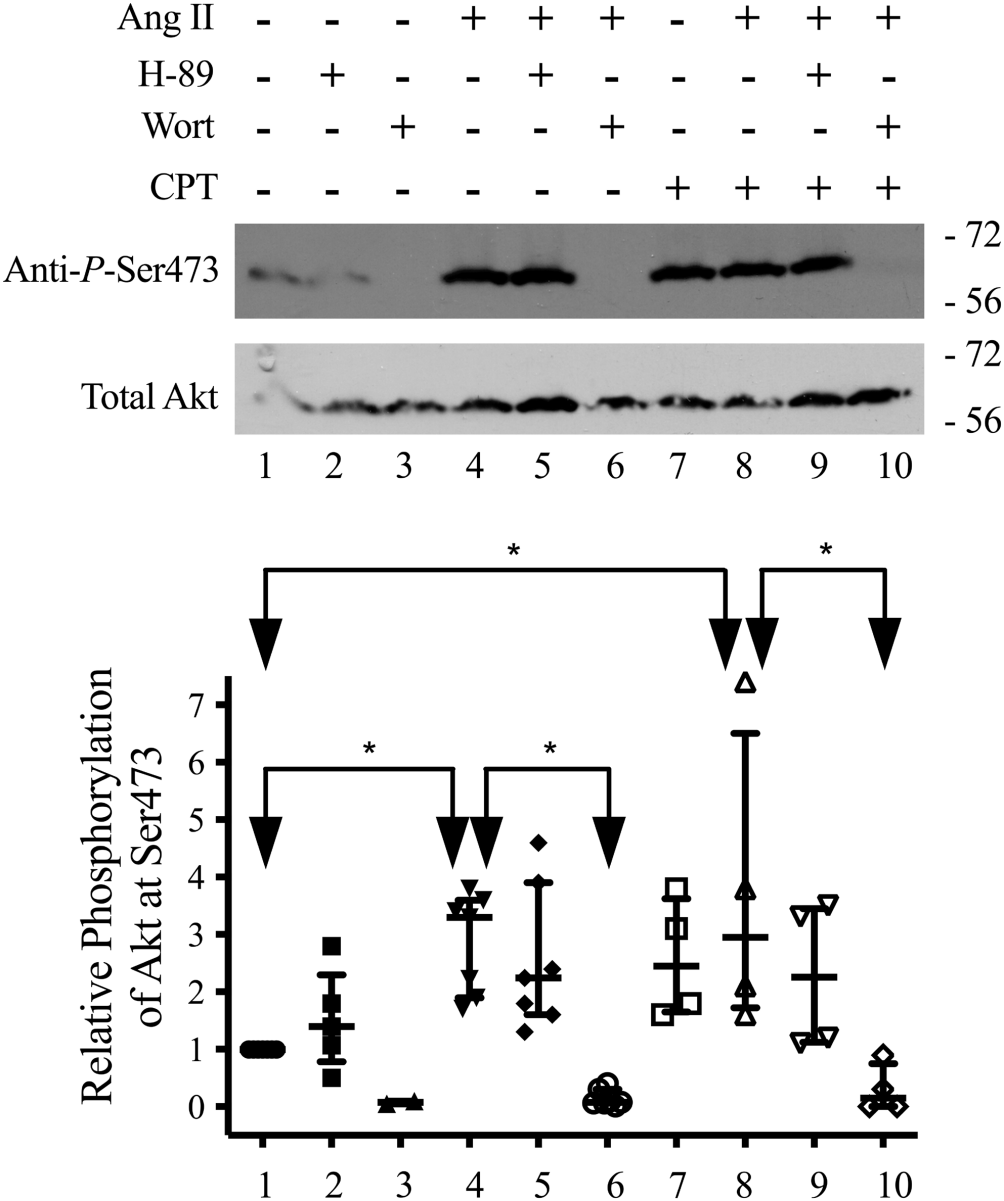
The effect of 100 pM Ang II on the phosphorylation of AKT at S473 in the presence and absence of 10 μM H‐89 and 0.2 μM wortmannin in α‐1.wild‐type cells; *n* = 4. The horizontal lines are the median and interquartile range. Each lane contained similar amounts of total AKT as shown in the lower blot that analyzed the same samples and was run at the same time as the upper blot showing anti‐P‐Ser473. The arrows labeled with “*” show which samples are different from each other as analyzed by a one‐way ANOVA followed by Bonferroni's post‐hoc multiple comparison.

As a test of our hypothesis, we determined if Ang II activated AKT as shown by the ability of Ang II to increase the phosphorylation of AKT at S473. We found that Ang II did increase phosphorylation of AKT at S473 compared to control (Figure 6, lane 1 vs. lane 4). This increase was inhibited by wortmannin (Figure 6, lane 6), but not H‐89 (Figure 6, lane 5).

To determine if activation of AKT could be sensitive to changes in cAMP, we tested if CPT‐2Me‐cAMP (CPT), a synthetic activator of EPAC (exchange factor directly activated by cAMP), at 10 μM for 20 min also increased the phosphorylation of AKT at S473 (Figure 6, lane 1 vs. lane 7). This increase occurred in the presence of H‐89 (Figure 6, lane 9), but not in the presence of wortmannin (Figure 6, lane 7 vs. lane 10). Also, to determine if phosphorylation of S938 could be sensitive to changes in cAMP independent of PKA, we tested if CPT treatment significantly increased the phosphorylation of the Na,K‐pump at S938 in α‐1.wild‐type cells as analyzed by a paired t‐test. Compared to control the stimulation was 2.64‐fold. The values in 4 independent samples were: 1.74, 2.13, 4.72, and 1.98‐fold.

## 4 DISCUSSION

The results of this study support our hypothesis that acute Ang II‐dependent activation of the rat kidney‐pump in the proximal tubule is mediated at least in part via increased phosphorylation of the Na,K‐pump at S938 via a PI3K/AKT(PKB)‐dependent pathway. Our data show that preventing phosphorylation of S938 blocks the ability of Ang II to acutely stimulate the Na,K‐pump and increase the net movement of sodium from the apical to basolateral side of differentiated cultured proximal tubules. Previous studies on the same cells not differentiated into distinct apical and basolateral membranes showed that phosphorylation of S938 was required for Ang II to rapidly (<5 min) increase the amount of Na,K‐pump in the plasma membrane and thereby stimulate Na,K‐pump activity (Massey et al., 2016), but did not prove that Na,K‐pump activation increased net sodium transport across the cells as occurs in vivo. Significantly, the results of the present study also support the novel conclusion that S938 is phosphorylated by AKT by at least two mechanisms. The first is Ang II mediated activation of PI3K, which in turn activates AKT to phosphorylate S938 and acutely stimulate Na,K‐pump activity. The other proposed novel mechanism, likely unrelated to Ang II, is that cAMP binds to EPAC, which in turn activates PI3K to stimulate AKT to phosphorylate the Na,K‐pump at S938 with yet to be documented effects on Na,K‐pump activity.

The identification of AKT‐dependent signaling pathways that can phosphorylate the Na,K‐pump at S938 is significant because all mammalian cells have a Na,K‐pump in the plasma membrane to control ion gradients, which are fundamental to cell homeostasis. All have a conserved site of PKA phosphorylation at S938 (Beguin et al., 1994; Fisone et al., 1994) or its equivalent,^1^ consistent with S938 being a major site of regulation (Poulsen et al., 2010). Nevertheless, there have been long‐standing questions as to whether PKA changes the phosphorylation at S938 under physiological conditions (Sweadner & Feschenko, 2001) and how changes in cAMP, which stimulate PKA, regulate Na,K‐pump activity (Mordasini et al., 2005; Therien & Blostein, 2000). Thus, our data supporting the conclusion that the rat kidney Na,K‐pump can also be phosphorylated at S938 via PI3K/AKT could be broadly relevant to human physiology. Likewise, our data which support the conclusion that cAMP/EPAC can increase S938 phosphorylation via AKT independent of PKA and Ang II provides additional insight into how the Na,K‐pump could be regulated. Our data also support the conclusion that the PI3K/AKT pathway inhibits PKA‐mediated phosphorylation of S938 and vice versa. Thus, our results help to define mechanisms relevant to understanding how Ang II‐dependent stimulation of sodium transport across proximal tubules increases blood pressure in humans and provide evidence for a novel pathway by which cAMP could control phosphorylation at S938 independent of Ang II.

The specific evidence supporting our hypothesis is that 10 pM Ang II acutely stimulated Na,K‐pump activity in α‐1.wild‐type cells, but not in α‐1.S938A cells. Second, 10 pM Ang II increased the phosphorylation of the Na,K‐pump at S938 in whole cell lysates. Third, Ang II‐dependent increases in phosphorylation at S938 observed in the presence of H‐89 were blocked by wortmannin (Figure 4), a selective inhibitor of PI3K at the concentration used in our study (Bain et al., 2007). Ang II‐dependent increases in phosphorylation at S938 were not inhibited by H‐89 (Figure 4), a selective inhibitor of PKA that does not inhibit PI3K under our experimental conditions (Chijiwa et al., 1990). Fourth, Ang II activated AKT, as shown by its ability to increase the phosphorylation of AKT (Figure 6). This particular result strongly supports our hypothesis because the residues surrounding S938 fit the substrate motif for phosphorylation by AKT (Obata et al., 2000). The Ang II‐dependent increase in AKT phosphorylation was blocked by wortmannin, and not H‐89.

**FIGURE 7:**
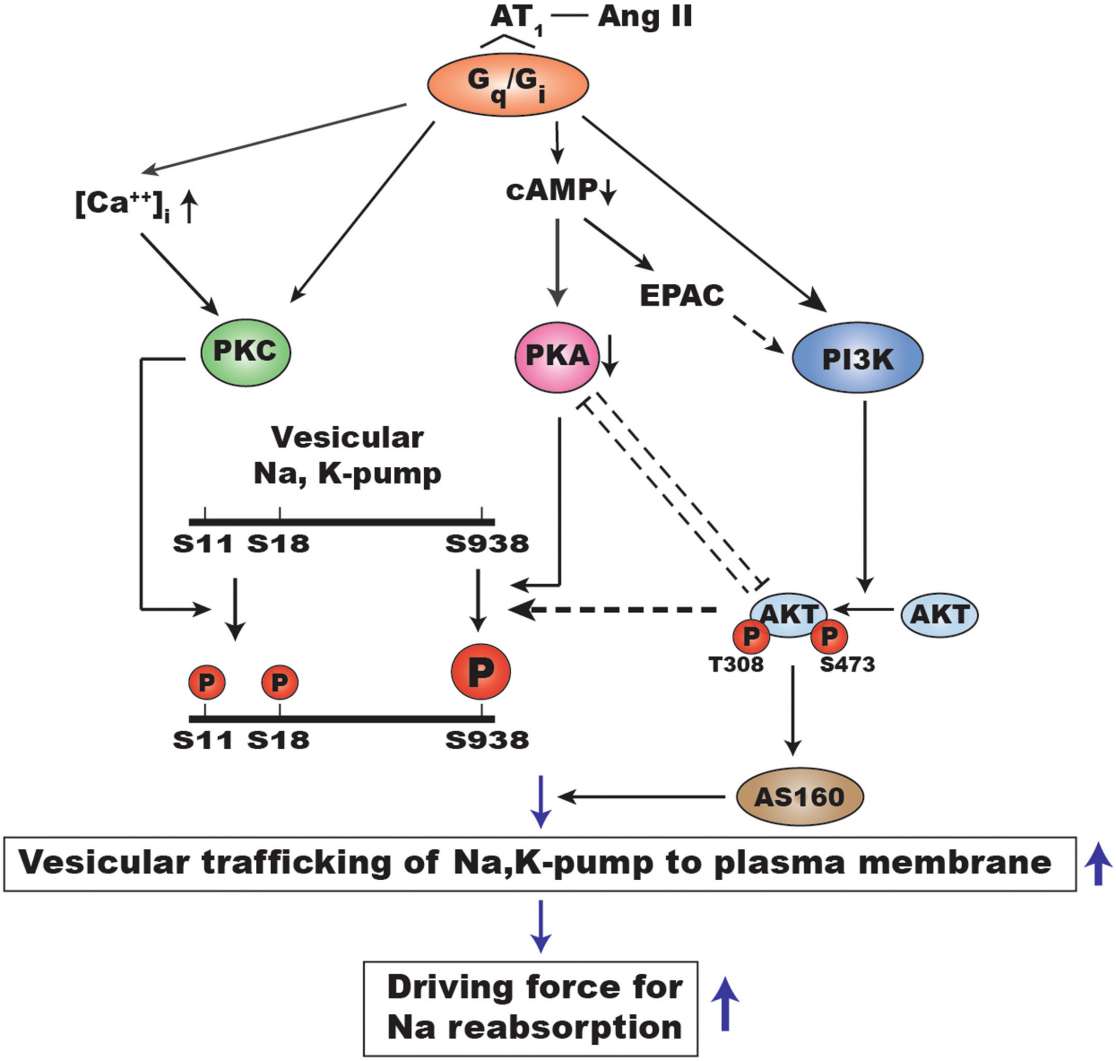
How ang II increases the phosphorylation of vesicular Na,K‐pumps at S938 and thereby stimulates Na,K‐pump activity and the transcellular transport in proximal tubules. The solid lines show pathways already shown by others. The dashed lines represent proposed pathways based on the results of the present study. Note that Na,K‐pumps in the plasma membrane of proximal tubules are not shown in the diagram. Also, the α‐subunit of the human kidney Na,K‐pump has sites homologous to S11 and S938, but not S18 (Poulsen et al., 2010).

We propose that PI3K/AKT (PKB) mediated increases in phosphorylation at S938 play a significant role in the mechanism by which Ang II acutely stimulates Na,K‐pump activity in the proximal tubule (Figure 7). It has been known that the ability of Ang II to increase the trafficking of the rat kidney Na,K‐pump to the plasma membrane required multiple events: PKC‐dependent phosphorylation of S11 and S18 (Efendiev et al., 2003), phosphorylation of S938 (Massey et al., 2016), and the binding of adaptor protein‐1 (AP‐1) to the α‐subunit of the Na,K‐pump (Efendiev et al., 2008). Also, in the context of renal ischemia, trafficking of the Na,K‐pump to the plasma membrane is partially dependent on AKT phosphorylation of its substrate AS160 (Alves et al., 2015). Inhibiting any one of these events could reduce the ability of Ang II to stimulate Na,K‐pump activity in the proximal tubule via increased trafficking to the plasma membrane. In vivo PKA activity and the resulting inhibition of AKT would be reduced, not by H‐89, but rather by the decline in cAMP levels that occurs when Ang II binds to the AT_1_ receptor (Thekkumkara et al., 1998) (Figure 7). We would thus predict that a decline in cAMP would coordinate with a reduction in the extent to which AKT inhibits PKA (Figure 7). Overall, because both PKA and AKT can phosphorylate S938, the two pathways would cooperate in the regulation of the trafficking of the Na,K‐pump to regulate its activity.

Based on our results we propose that AKT phosphorylates Na,K‐pumps that are in intracellular vesicles and PKA phosphorylates Na,K‐pumps that are in the plasma membrane. Using our model of reciprocal inhibition between AKT and PKA (Figure 7), one would predict that Ang II mediated activation of AKT would increase the phosphorylation of S938 in vesicles and decrease S938 phosphorylation in the plasma membrane. Also, one would predict that adding wortmannin to inhibit PI3K would not necessarily block increased phosphorylation of S938 as measured in cell lysates, because the addition of wortmannin to inhibit AKT released AKT inhibition of PKA, which then phosphorylated S938 in the plasma membrane. Thus, reciprocal inhibition accounts for the observation that adding either H‐89 alone (Figure 4, lane 2 vs. lane 1) or wortmannin alone (Figure 4, lane 3 vs. 1) increased the level of S938 phosphorylation measured in whole cell lysates (Figure 4). It also accounts for the observation that it required both H‐89 and wortmannin to inhibit Ang II‐dependent phosphorylation of S938 as observed in whole cell lysates (Figure 4). In conclusion, we suggest that wortmannin alone blocked Ang II‐dependent stimulation of Na,K‐pump activity (Figure 3b), because it inhibited both PI3K‐mediated activation of AKT, which has been shown to promote the trafficking of the Na,K‐pump to the plasma membrane through its phosphorylation of AS160 (Alves et al., 2010, 2015) and AKT phosphorylation of S938 in vesicles.

We note that the ouabain‐sensitive change in Isc in α‐1.S938 cells in the absence of Ang II (Figure 3c) is about 1/2 of that in α‐1.wild‐type cells (Figure 3a). On the other hand, Na,K‐pump activity in α‐1.S938 cells measured as ouabain‐sensitive Rb uptake per mg protein under control conditions is the same as in α‐1.wild‐type cells (Massey et al., 2016), which shows that the two cell systems have comparable amounts of Na,K‐pump. Perhaps the reduction in the ouabain‐sensitive current in α‐1.S938A cells is due to the mutation itself which increases a leak pathway through the Na,K‐pump (Poulsen et al., 2010) that results in a reduction in the ouabain‐sensitive current per Na,K‐pump.

As specified above the interpretation of our phosphorylation results in terms of signaling pathways is based on the expectation that we have used wortmannin under conditions that it selectively inhibits PI3K and H‐89 under conditions that it selectively inhibits PKA. On the other hand, exposing murine cortical collecting duct cells to 100 nM wortmannin for hours reduces epithelial integrity, with some effects observed in as little as 30 min (Mansley & Wilson, 2010). In our experiments, proximal tubule cells were preincubated with 200 nM wortmannin for 15 min; Na,K‐pump activity was then measured during the next 5 min period. We found that the activity of the Na,K‐pump in the absence of Ang II was greater in the presence of wortmannin than in its absence (Figure 3), which is inconsistent with there being significant membrane damage during our experiments. Nevertheless, the concentrations of wortmannin that we used in our experiments could have inhibited myosin light chain kinase (Bain et al., 2007; Mansley & Wilson, 2010). Therefore, although we cannot entirely rule out all such off‐target effects of either H‐89 or wortmannin, we conclude that the use of these two inhibitors support our underlying hypothesis and lay the foundation for further defining the proposed signaling pathways.

Results consistent with aspects of the signaling model shown in Figure 7 have been observed by others. For instance, activation of the AT_1_ receptor stimulates a PI3K/AKT (protein kinase B) pathway in rat proximal tubule cells (Sugawara et al., 2021) and in smooth muscle (Dugourd et al., 2003), which includes EPAC (Takahashi et al., 1999). Others have shown that AKT is a key mediator of the PI3K signaling pathway (Fayard et al., 2010). Also, sequential activation of PI3K/EPAC in LLC‐PK1 cells, a proximal tubule cell line, inhibits PKA activity (Peruchetti et al., 2011). Furthermore, activation of the AT_1_ receptor in a mouse mesangial cell line increases phosphorylation of AKT at T308 in less than a minute via a cAMP‐dependent PI3K‐AKTpathway. This pathway is blocked by 2,5 DOA, an inhibitor of AKT, but not by H‐89 (Bu et al., 2011). Activation of AKT regulates the trafficking of the Na,K‐pump in the kidney via AS160 (Alves et al., 2010, 2015) and mediates insulin‐stimulated trafficking of the Na,K‐pump in alveolar epithelial cells (Comellas et al., 2010). Furthermore, it has been demonstrated that phosphorylation of S955, a homologous site of PKA phosphorylation in the closely related H, K‐ATPase, helps control the surface expression of the H, K‐ATPase in the colon (Codina et al., 2006). Our observation that CPT increases the phosphorylation at S938 shows that cAMP acting via EPAC can increase the phosphorylation of S938 independent of binding to and activating PKA. Thus, there is likely a mechanism by which cAMP controls phosphorylation at S938 and Na,K‐pump activity in the proximal tubule in addition to direct phosphorylation via PKA.

Our finding that 10 pM Ang II stimulated rat Na,K‐pump activity, whereas 100 pM had no effect on Na,K‐pump activity, fits the well‐established pattern that low pM concentrations of Ang II stimulate, intermediate concentrations have no effect, and higher concentrations of Ang II inhibit both Na,K‐pump activity (Bharatula et al., 1998) and sodium reabsorption in rodent proximal tubules (Harris & Young, 1977). Yet, both 10 and 100 pM Ang II increased the phosphorylation at S938 measured in cell lysates (Figure 5). In this regard it is important to recognize that acute stimulation of Na,K‐pump activity is not expected to be a linear function of any single mechanism for its regulation, including phosphorylation at S938. As outlined above, acute stimulation of rat kidney Na,K‐pump activity by Ang II requires activation of PKC to phosphorylate the Na,K‐pump at S11 and S18, and activation of PI3K to induce stimulation of AKT that then phosphorylates S938 on the Na,K‐pump and AS160 (Figure 7). As the concentration of Ang II increases, so does the extent to which Ang II increases the concentration of intracellular free calcium (Madhun et al., 1991), which can inhibit Na,K‐pump activity (Okafor et al., 1997; Yingst, 1988).

Based on the crystal structure of the Na,K‐pump, it has been predicted that increased phosphorylation at S938 should shift the Na,K‐ATPase toward its E2 conformation and decrease its affinity for intracellular sodium (Einholm et al., 2016). Such a decrease could be part of the mechanism by which higher concentrations of Ang II inhibit the activity of the rat Na,K‐pump in the plasma membrane. Consistent with this idea, we have shown that treating α‐1.wild‐type cells with 10,000 pM Ang II, which would be expected to inhibit the transport activity of the rat Na,K‐pump (Bharatula et al., 1998), both increases phosphorylation at S938 in cell lysates and increases the affinity for digoxin in α‐1S11A/S18A cells (Massey et al., 2012). An increase in the affinity for digoxin, which binds to the E2 conformation of the Na,K‐pump, would in turn be consistent with a decrease in the affinity of the Na,K‐pump for intracellular sodium.

Other potential effects of Ang II on the kinetics of the Na,K‐pump include the observation that incubating rat proximal tubules with Ang II increases the apparent affinity of the Na,K‐pump for intracellular sodium (Yingst et al., 2004). An apparent increase could be explained by a shift in the E1/E2 poise of the Na,K‐pump toward E1 mediated by increased phosphorylation of sites in the first 32 amino acids of the N‐terminus (Yingst et al., 2008). Alternatively, the apparent affinity for sodium could be increased via dephosphorylation of S938 on Na,K‐pumps that have recently been trafficked to the plasma membrane. In our studies in rat proximal tubules we documented that Ang II increased the phosphorylation of the Na,K‐pump in at least three phosphopeptides (Yingst et al., 2004). We now suggest that one of these could have been phosphorylated S938 and the other two were likely S11 and S18 (Efendiev et al., 2003).

In humans, increasing Ang II from 10^−10^ to 10^−6^ M progressively stimulates sodium transporters (NHE3 and NBCe1) in the proximal tubule (Shirai et al., 2014). In both humans and rats, the effects of Ang II on sodium transport in the proximal tubule are mediated via the AT_1_ receptor, which in turn activates PI3K/AKT signaling. In addition, both rat and human forms of the kidney Na,K‐pump contain the same site of PKA phosphorylation. Thus, phosphorylation of the Na,K‐pump at S938 could be part of the mechanism by which 10 pM Ang II stimulates sodium reabsorption in both species.

In summary, we propose that phosphorylation of S938 by AKT activated by PI3K is part of the mechanism by which Ang II acutely stimulates the Na,K‐pump in both rat and human proximal tubules. Ang II‐dependent increases in sodium reabsorption in the proximal tubule increase resting blood pressure (Gurley et al., 2011; Li et al., 2011). Insulin also stimulates PI3K/AKT signaling (Zheng et al., 2005) and stimulates Na,K‐pump activity in the rat proximal tubule (Feraille et al., 1999). Therefore, one would predict that each hormone could influence the effect of the other on Na,K‐pump activity, sodium reabsorption, and blood pressure via PI3K/AKT signaling and the regulation of Na,K‐pump phosphorylation at S938. In addition, we suggest that the binding of cAMP to EPAC activates PI3K to stimulate AKT to phosphorylate Na,K‐pump at S938, which would represent an additional mechanism by which the Na,K‐pump could be regulated.

## AUTHOR CONTRIBUTIONS

DRY and RRM conceived the project. FSH, SA, CR, and RRM performed experiments. KJ helped design figures. DRY with input from RRM drafted the manuscript. All authors approved the final version.

## FUNDING INFORMATION

This work was supported by the National Institutes of Health [grant numbers R01‐DK60752 (to D.Y.) and R01‐CA81150 (to R.R.M.)]. Confocal microscopy was performed through the Wayne State University and Karmanos Cancer Institute Microscopy, Imaging and Cytometry Resources Core, which is supported in part by National Institutes Health center grant P30 CA22453.

## CONFLICT OF INTEREST

The authors declare that they have no conflict of interest.

## Endnote

^1^
S938 is homologous to S943 in the human a‐1 subunit of the kidney Na,K‐pump.
